# Case report: A girl with witnessed sleep apnea

**DOI:** 10.3389/fneur.2023.1337236

**Published:** 2024-01-11

**Authors:** Shuai Wu, Waner Wang, Fang Han, Liyue Xu

**Affiliations:** ^1^Division of Sleep Medicine, Peking University People’s Hospital, Beijing, China; ^2^Division of Sleep Medicine, Peking University International Hospital, Beijing, China

**Keywords:** Pfeiffer syndrome, obstructive sleep apnea, polysomnography, central sleep apnea, positive airway pressure

## Abstract

**Introduction:**

Pfeiffer syndrome is a rare genetic disorder characterized by craniosynostosis, broad thumbs and big toes, and partial syndactyly of the hands and feet. This case report presents the case of a girl diagnosed with type 2 Pfeiffer syndrome who experienced severe obstructive sleep apnea (OSA).

**Case report:**

The patient had been using an oropharyngeal airway since the age of 4 months due to snoring and witnessed apnea during sleep. At 11 months old, she was referred to our sleep clinic because of growth limitation and gross motor ability issues. Polysomnography (PSG) showed severe obstructive hypopnea before any treatment, and revealed severe central sleep apnea with the oropharyngeal airway in place. Positive airway pressure (PAP) therapy was initiated, which improved both her sleep and gross motor ability.

**Conclusion:**

This case report emphasizes the importance of thorough sleep studies for diagnosing sleep and breathing disorders in Pfeiffer syndrome patients and highlights the effectiveness of PAP therapy in managing these conditions.

## Introduction

Pfeiffer syndrome, caused by FGFR1 or FGFR2 mutations, ranges from mild Type 1 with normal development to more severe Types 2 and 3. Pfeiffer syndrome presents in more severe forms as Types 2 and 3, often associated with neurological complications. Premature fusion of skull bones in these types can impede brain growth, resulting in developmental delays and other neurological issues. Type 2 and 3 individuals may experience ankylosis in the elbow or other joints, limiting mobility, as well as facial and airway abnormalities that can lead to life-threatening breathing difficulties. Type 2 is distinguished by the presence of a cloverleaf-shaped head due to extensive skull bone fusion, while Type 3 also exhibits severe symptoms and a poor prognosis (1, 2).

Patients often experience upper airway obstruction, necessitating PSG monitoring and potentially craniofacial reconstructive surgery or other interventions like PAP therapy or nasopharyngeal airway to manage symptoms (3–5).

We report a case of an 11-month-old girl with type 2 Pfeiffer syndrome and severe obstructive sleep apnea (OSA), who developed severe central sleep apnea while being treated with an oropharyngeal airway. The transition to positive airway pressure (PAP) therapy resolved the sleep apnea and improved her gross motor skills, thereby setting the stage for the Le Fort III osteotomy.

## Case report

The patient was born at 35-week gestation and had a low birth weight of 2.32 kg. After birth, she was diagnosed with premature closure of craniosiosutures and cranial hypertension. DNA testing revealed a mutation in the *FGFR2* gene, confirming the diagnosis of Pfeiffer syndrome. Her parents and grandparents were healthy.

During the neonatal period, the patient started snoring and experienced episodes of witnessed apnea. She also displayed frequent sleep disruptions, easy awaking, and irritability upon awakening. Parents also noted that her eyes remained open during sleep. At 4 months old, she underwent adenoidectomy without any improvement. Subsequently, she was admitted to intensive care unit due to pneumonia. Prior to discharge, the pediatrician and intensivist fitted her with an oropharyngeal airway to treat the snore and sleep apnea.

Although the oropharyngeal airway helped alleviate the snoring, her eyes remained open during sleep, and she exhibited limited gross motor ability. She could not raise her head and turn around when she was 11 months old. She was referred to our sleep clinic by a maxillofacial surgeon. A multidisciplinary medical team, including specialists in brain surgery, maxillofacial surgery, thoracic surgery, anesthesiology, and sleep medicine, was assembled to provide comprehensive care for this patient. Based on the literature review, the team determined that LeFort III osteotomy would be the most appropriate treatment option. However, the team decided to delay the surgery until the patient reached one and a half years of age. As of the literature review before the patient’s visit, the youngest age for patients undergoing Le Fort III osteotomy was 2 years old (6) whereas our patient was only 11 months old. Additionally, her growth and motor abilities were limited. Initiating the surgery at an earlier age may result in a higher risk of failure and complications. Thus, the medical team decided to delay the surgery until she was 1.5 years old. Instead, the use of PAP therapy was intended to improve her sleep apnea and support her growth.

**Figure 1:**
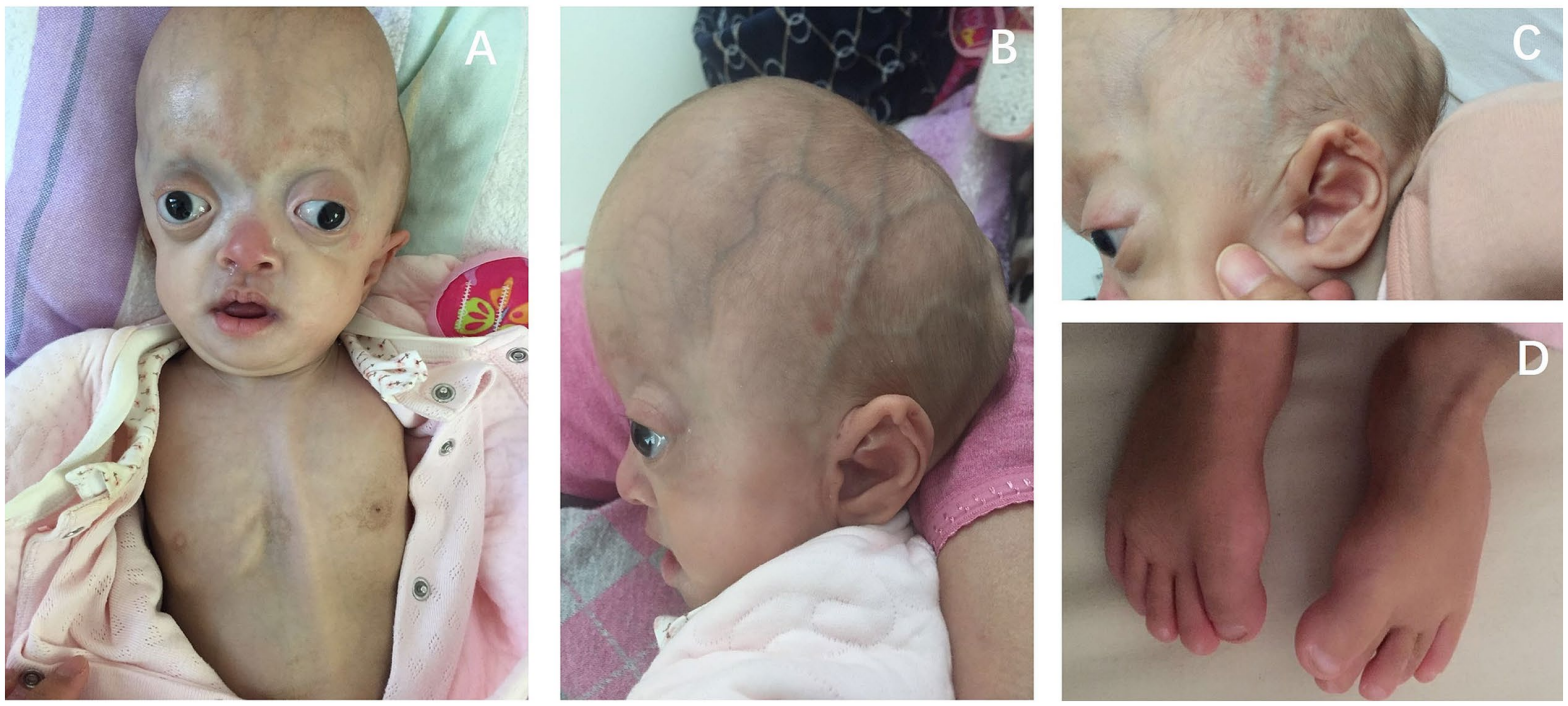
Physical features of this girl showed the child’s wide and flat skull deformity, large eye slits, wide interocular distance, protruding eyeballs, bilateral exotropia, absence of upper eyelid ptosis, small and low nasal bridge **(A)**. The forehead is high and prominent, the posterior part of the skull is flat, and the skull has a cloverleaf appearance, with a central arched elevation and bilateral depressions **(B)**. The ears are low-set, with bilateral closed external ear canals **(C)**. The big toe is wide and malformed **(D)**.

Upon evaluation at sleep clinic, the patient’s physical examination revealed craniofacial and limb deformities (Figure 1). The patient exhibited limited head mobility and difficulty in turning around. Paradoxical breathing was observed after falling asleep (Supplementary Video S1). Cardiac echocardiography did not reveal any abnormalities. Head CT scan indicated no presence of Chiari malformation.

**Figure 2:**
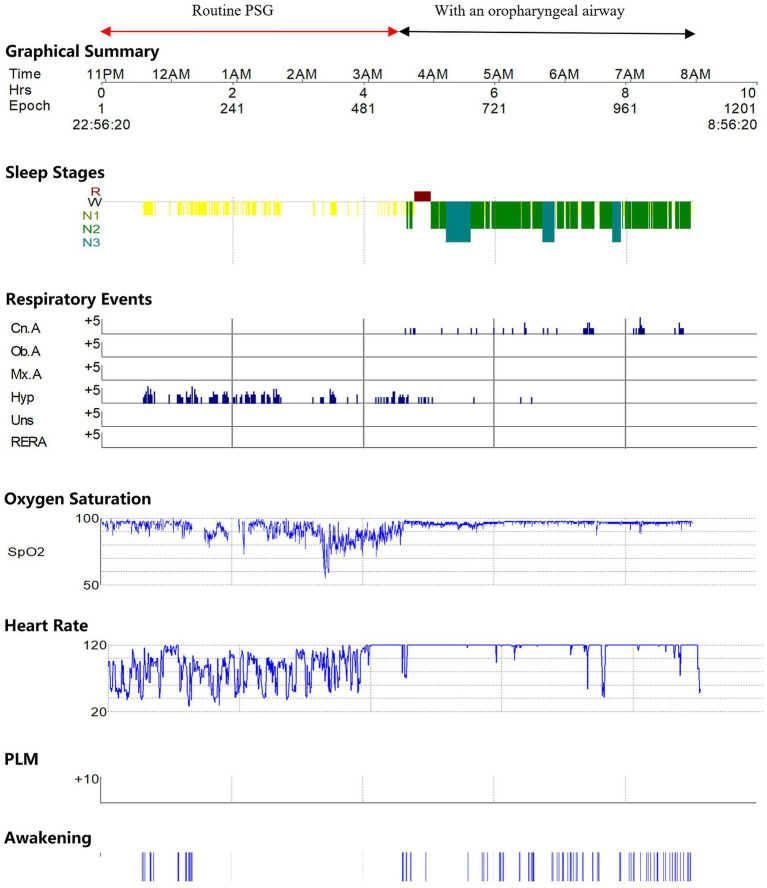
Illustration of PSG report showed that in the first half of the monitoring, the girl did not enter deep sleep, experienced frequent episodes of obstructive hypopnea, and had frequent drops in oxygen saturation accompanied by significant heart rate variability. In the second half of the monitoring with an oropharyngeal airway, the girl could enter deep sleep, significantly reducing obstructive hypopnea. However, central sleep apnea events were observed.

**Figure 3:**
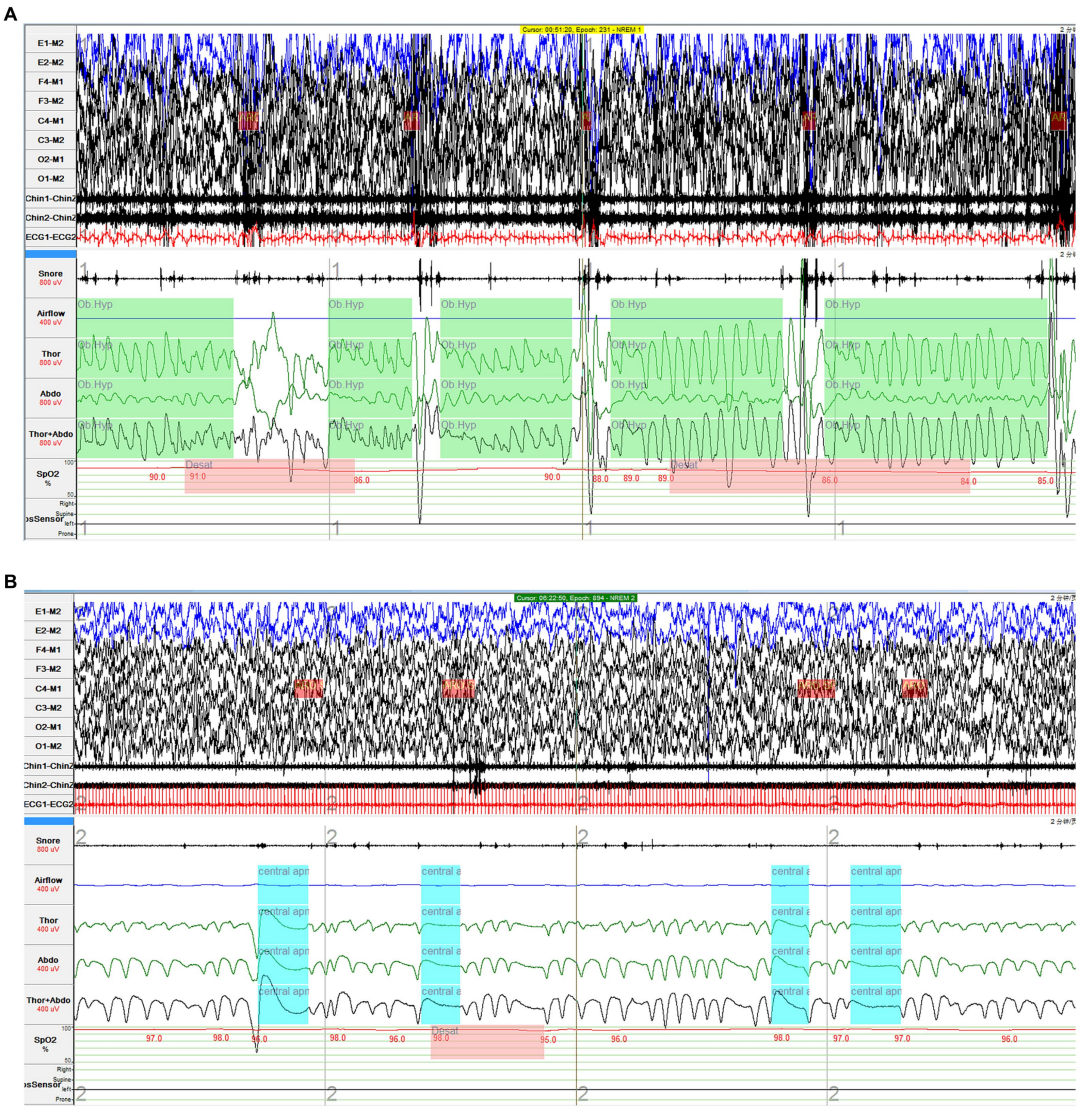
Obstructive hypopnea and central sleep apnea in the diagnostic PSG. **(A)** In the first half of the night, the routine PSG monitoring showed that the girl experienced frequent obstructive hypopnea with paradoxical breathing. **(B)** In the second half of the night, severe central sleep apnea events were observed after she wore an oropharyngeal airway.

The girl underwent a split PSG recording (Figure 2). From 23:25 to 03:26 am, the regular PSG revealed a severe AHI of 117.7 events/h, consisting solely of obstructive hypopnea (Table 1; Figure 3A). Her average oxygen saturation (SpO2) was 89%, and her sleep structure was significantly disrupted, with 79 min of N1 sleep. From 03:26 a.m. to 07:56 a.m., she utilized an oropharyngeal airway device. The second part of the PSG revealed a reduction in the obstructive events index to 3.7 events per hour. However, the central sleep apnea index was 11.9 events per hour (Table 1; Figure 3B), resulting in an overall AHI of 15.6 events per hour. Based on the PSG findings, the girl was diagnosed with: (1) severe OSA associated with type 2 Pfeiffer syndrome, and (2) severe central sleep apnea syndrome with the use of an oropharyngeal airway.

**Table 1:** Diagnostic PSG.

	Whole night	Routine PSG (the first half night)	With an oropharyngeal airway (the second half of the night)
Timing	23:25–07:56	23:25–03:26	03:26–07:56
Total recording time (min)	511	241	270
Total sleep time (min)	325.5	79	246.5
Sleep efficiency	63.7%	32.8%	91.3%
N1 percentage	29.2%	100%	6.5%
N2 percentage	53.5%	0	70.6%
N3 percentage	12.7%	0	16.8%
REM percentage	4.6%	0	6.1%
AHI (events/h)	40.3	117.7	15.6
Obstructive sleep apnea index (events/h)	0	0	0
Obstructive hypopnea index (events/h)	31.4	117.7	3.7
Central sleep apnea index (events/h)	9.0	0	11.9
Mean oxygen saturation	94.3%	89%	96%
ODI3 (events/h)	21.2	54.7	10.5
Arousal index (events/h)	14.0	12.9	14.4

We initiated the PAP treatment for this patient. She slept well with eyes closed at a CPAP pressure of 8.5 cmH_2_O. According to the parents, the patient exhibited increased happiness and laughter. Notably, her gross motor capacity improved as she was able to raise her head. We conducted a second manually pressure titration, adjusting the pressure to 11.5cmH_2_O because her parents reported snoring during PAP treatment. Subsequent PSG showed a residual AHI was 0.1 events per hour under CPAP 11.5 cm H_2_O. Following 6 months of PAP treatment, the patient had an average daily usage of 432 min per night, with 95.2% of days exhibiting usage time of at least 4 h.

After 6 months of continuous PAP treatment, the girl demonstrated significant improvements in her motor skills. She was able to turn over, sit up, and climb by the age of one and a half years. With these positive developments, the multidisciplinary medical team determined to proceed with mid-face surgery. The girl subsequently underwent a LeFort III osteotomy. Following the surgery, she no longer experienced snoring. PSG without EEG indicated an AHI of 1.2 events per hour. As result, the girl discontinued PAP treatment after the surgery.

## Discussion

Craniosynostosis is a congenital disorder characterized by premature fusion of the cranial sutures, which restricts the normal skull, brain, and facial growth. This condition is associated with a higher incidence and more severe clinical manifestations of OSA. Studies have shown that in infants with Pfeiffer syndrome and symptoms of excessive sleepiness or sleep disturbances, the prevalence of OSA is approximately 72.7%, with 45.5% classified as severe OSA (7). The high occurrence of OSA in these infants may be attributed to facial skeletal abnormalities causing upper airway narrowing or increased intracranial pressure.

OSA can have significant implications for children, including cognitive and behavioral problems, delayed growth and development, and even pulmonary arterial hypertension. The risk is further elevated in patients with craniosynostosis (8). Therefore, regular screening for OSA is necessary in patients with craniosynostosis, and PSG is the golden diagnostic method. Because the patient had never undergone a sleep study previously, the sleep team initially planned to conduct two separate nights of PSG to evaluate her baseline sleep and the efficiency of the oropharyngeal airway. However, the patient found it very difficult to undergo the PSG due to sensitivity in her head, resulting in irritability when the electrodes were placed. Her severe obstructive sleep apnea led to frequent awakenings, and after 3 h of recording, the medical team decided to proceed with the split study. Subsequently, utilizing the oropharyngeal airway under PSG monitoring allowed us to assess the treatment efficiency, providing evidence to support the transition to PAP treatment.

When it comes to treatment, adenotonsillectomy is the most common intervention for OSA in pediatric patients. Other treatment options include midface advancement surgery, PAP, tracheostomy, and nasal pharyngeal airway (8, 9).

Nasopharyngeal airway can be also used to treat OSA patients who tolerate it well, regardless of whether adenotonsillectomy is performed or not (8). In this case, although oropharyngeal airway significantly reduced obstructive hypopnea and improved oxygen saturation, there were central sleep apneas occurring at a rate of 11.9 per hour. Although the patient did not exhibit Chiari malformation, central sleep apnea emerged when using the oropharyngeal airway. The specific mechanism behind this remains unclear. The authors have the following speculations: (1) The patient experienced unstable sleep when using the oropharyngeal airway. While the oropharyngeal airway significantly reduced obstructive events, its invasiveness may have compromised comfort. Our observations indicate a lack of N3 stage in patient when using the oropharyngeal airway, supporting that the patient experienced disturbed sleep and could not enter to slow wave sleep under oropharyngeal airway treatment. (2) The occurrence of central sleep apneas may be attributed to an increased loop gain and resulting ventilatory instability following the use of the oropharyngeal airway (10). It is regrettable that we did not monitor the patient’s continuous CO2 levels, which could have helped distinguish between central apnea related to high or low carbon dioxide. CPAP therapy, a non-invasive treatment, by providing continuous positive pressure, maintained airway patency and prevented inhibitory reflexes during airway closure, effectively reducing loop gain and enhancing ventilatory stability, thereby improving the sleep structure and central sleep apnea (11). Ultimately, this case underscores the importance of not relying solely on pulse oximetry to assess the effectiveness of apnea treatment in complex cases; evaluating respiratory movement and sleep structure is equally critical.

Additionally, adenotonsillectomy is less efficacious where midfacial hypoplasia is the primary cause of OSA in infants with craniofacial synostosis. Midface advancement surgery, particularly the standard Le Fort III osteotomy, is the preferred method for addressing the complications in patients with craniofacial deformities, including OSA (6, 12). Le Fort III osteotomy is a maxillofacial surgical procedure primarily aimed at correcting midfacial hypoplasia. The procedure entails incisions near the forehead, temples, and nose, detaching the midface from the skull, repositioning, and securing it in a new alignment (5). In the reported case, postoperative snoring disappeared, and the follow-up AHI was 1.2 events/h. However, long-term follow-up is still necessary to confirm the efficacy, and the possibility of OSA recurrence cannot be ruled out.

Although PAP therapy is generally safe in the vast majority of cases, it is worth noting that PAP therapy can present significant challenges in infants, especially those with craniofacial deformities, despite early attempts to employ PAP therapy for their treatment (13). For example, PAP therapy may potentially irritate the conjunctiva, leading to tearing and discomfort in the eyes, as well as skin breakdown and nasal injuries, long-term usage could even result in midface retrusion (14) These complications are more likely to occur when the mask fit is inadequate. In our case, the parents manually modified the headgear to ensure a proper fit for the infant’s head shape. In addition to using a suitable mask and headgear and adjusting the treatment pressure appropriately, successful PAP therapy also requires efforts from the medical team and family members to assist and encourage the infant in adapting to and tolerating the treatment.

Our report emphasizes the importance of PSG to assess the severity of sleep disorders in pediatric patients, especially those with craniofacial anatomical abnormalities. Insufficient development of the facial skeleton in Pfeiffer syndrome, resulting in a narrowed upper airway or increased pressure, caused OSA. The use of oropharyngeal airways can help alleviate obstructive events. However, as observed in our case, central events may occur. Midface advancement surgery is the preferred treatment. When surgery is not feasible, PAP therapy can effectively alleviate respiratory events, including central sleep apneas, and improve physical function, providing a better opportunity for surgical intervention. In fact, in this case, we observed an improvement in the patient’s neurocognitive function following PAP therapy. Similarly, research suggests that treating pediatric OSA can improve ADHD symptoms (15) as well as neurocognitive performance in patients with Pierre Robin sequence (16). Although the specific mechanism remains unclear, we speculate that it is likely associated with the enhancement of sleep quality and the reduction of hypoxia.

## Conclusion

In summary, our case of an 11-month-old girl diagnosed with type 2 Pfeiffer syndrome and severe OSA underscores the significant value of PAP therapy, the use of PAP treatment not only provided a crucial interim solution while awaiting surgical intervention but also demonstrated a marked improvement in her gross motor skills, highlighting the critical role of PAP therapy in managing complex craniosynostosis disorders with OSA.

## Data availability statement

The original contributions presented in the study are included in the article/Supplementary material, further inquiries can be directed to the corresponding author.

## Ethics statement

The requirement of ethical approval was waived by Peking University People’s Hospital for the studies involving humans due to its retrospective nature and the obtaining of informed consent: the report is a retrospective analysis of existing clinical data, involving no additional interventions or alterations to the standard care provided to the patient. The studies were conducted in accordance with the local legislation and institutional requirements. Written informed consent for participation in this study was provided by the participants’ legal guardians/next of kin. Written informed consent was obtained from the individual(s), and minor(s)’ legal guardian/next of kin, for the publication of any potentially identifiable images or data included in this article.

## Author contributions

SW: Writing – original draft. WW: Writing – original draft. FH: Writing – review & editing. LX: Conceptualization, Writing – review & editing.

## References

[1] Jezela-Stanek A Krajewska-Walasek M. Genetic causes of syndromic craniosynostoses. Eur J Paediatr Neurol. (2013) 17:221–4. doi: 10.1016/j.ejpn.2012.09.009 23062756

[2] Vogels A Fryns JP. Pfeiffer syndrome. Orphanet J Rare Dis. (2006) 1:19. doi: 10.1186/1750-1172-1-19, PMID: 16740155 PMC1482682

[3] Ahmed J Marucci D Cochrane L Heywood RL Wyatt ME Leighton SE. The role of the nasopharyngeal airway for obstructive sleep apnea in syndromic craniosynostosis. J Craniofac Surg. (2008) 19:659–63. doi: 10.1097/SCS.0b013e31816ae386, PMID: 18520380

[4] Fearon JA Rhodes J. Pfeiffer syndrome: a treatment evaluation. Plast Reconstr Surg. (2009) 123:1560–9. doi: 10.1097/PRS.0b013e3181a2057e, PMID: 19407629

[5] Nout E Cesteleyn LL Van Der Wal KG Van Adrichem LN Mathijssen IM Wolvius EB. Advancement of the midface, from conventional Le fort III osteotomy to Le fort III distraction: review of the literature. Int J Oral Maxillofac Surg. (2008) 37:781–9. doi: 10.1016/j.ijom.2008.04.006, PMID: 18486452

[6] Bannink N Nout E Wolvius EB Hoeve HL Joosten KF Mathijssen IM. Obstructive sleep apnea in children with syndromic craniosynostosis: long-term respiratory outcome of midface advancement. Int J Oral Maxillofac Surg. (2010) 39:115–21. doi: 10.1016/j.ijom.2009.11.021, PMID: 20056390

[7] Inverso G Brustowicz KA Katz E Padwa BL. The prevalence of obstructive sleep apnea in symptomatic patients with syndromic craniosynostosis. Int J Oral Maxillofac Surg. (2016) 45:167–9. doi: 10.1016/j.ijom.2015.10.003, PMID: 26602951

[8] Driessen C Joosten KF Bannink N Bredero-Boelhouwer HH Hoeve HL Wolvius EB. How does obstructive sleep apnoea evolve in syndromic craniosynostosis? A prospective cohort study. Arch Dis Child. (2013) 98:538–43. doi: 10.1136/archdischild-2012-302745, PMID: 23702437

[9] Kaditis AG Alonso Alvarez ML Boudewyns A Abel F Alexopoulos EI Ersu R. ERS statement on obstructive sleep disordered breathing in 1- to 23-month-old children. Eur Respir J. (2017) 50:1700985. doi: 10.1183/13993003.00985-2017, PMID: 29217599

[10] White DP. Pathogenesis of obstructive and central sleep apnea. Am J Respir Crit Care Med. (2005) 172:1363–70. doi: 10.1164/rccm.200412-1631SO 16100008

[11] Eckert DJ Jordan AS Merchia P Malhotra A. Central sleep apnea: pathophysiology and treatment. Chest. (2007) 131:595–607. doi: 10.1378/chest.06.2287, PMID: 17296668 PMC2287191

[12] Mixter RC David DJ Perloff WH Green CG Pauli RM Popic PM. Obstructive sleep apnea in Apert's and Pfeiffer's syndromes: more than a craniofacial abnormality. Plast Reconstr Surg. (1990) 86:457–63. doi: 10.1097/00006534-199009000-00011, PMID: 2385663

[13] Hoeve HL Joosten KF Van Den Berg S. Management of obstructive sleep apnea syndrome in children with craniofacial malformation. Int J Pediatr Otorhinolaryngol. (1999) 49:S59–61. doi: 10.1016/s0165-5876(99)00134-2 10577777

[14] Cielo CM Hernandez P Ciampaglia AM Xanthopoulos MS Beck SE Tapia IE. Positive airway pressure for the treatment of OSA in infants. Chest. (2021) 159:810–7. doi: 10.1016/j.chest.2020.08.020, PMID: 32805239 PMC7856529

[15] Urbano GL Tablizo BJ Moufarrej Y Tablizo MA Chen ML Witmans M. The link between pediatric obstructive sleep apnea (OSA) and attention deficit hyperactivity disorder (ADHD). Children. (2021) 8:824. doi: 10.3390/children8090824, PMID: 34572256 PMC8470037

[16] Bull MJ Givan DC Sadove AM Bixler D Hearn D. Improved outcome in Pierre Robin sequence: effect of multidisciplinary evaluation and management. Pediatrics. (1990) 86:294–301. doi: 10.1542/peds.86.2.294, PMID: 2371106

